# Draft Genome Sequencing of *Vibrio cholerae* O1 El Tor Isolates Collected in the Russian Federation from Imported Cholera Cases

**DOI:** 10.1128/genomeA.00624-14

**Published:** 2014-07-17

**Authors:** Konstantin V. Kuleshov, Sergey O. Vodop’ianov, Vladimir G. Dedkov, Mikhail L. Markelov, Anton V. Kermanov, Vladimir D. Kruglikov, Alexey S. Vodop’ianov, Ruslan V. Pisanov, Olga S. Chemisova, Alexey B. Mazrukho, Svetlana V. Titova, German A. Shipulin

**Affiliations:** a Federal Budget Institute of Science Central Research Institute for Epidemiology, Moscow, Russia; b Federal Government Health Institution Rostov-on-Don Plague Control Research Institute, Rostov-on-Don, Russia; c Institute of Occupational Health RAMS, Moscow, Russia

## Abstract

We report the draft genome sequencing of five *Vibrio cholerae* O1 El Tor clinical isolates collected in the Russian Federation from imported cholera cases in 2006, 2010, and 2012. In the initial phylogenetic analysis, one isolate clustered with the Haiti/Nepal-4 group.

## GENOME ANNOUNCEMENT

The geographical location of the Russia Federation and its integration with different countries are basic prerequisites for the importation of new strains of *Vibrio cholerae* from regions where the pathogen is endemic. The analysis of such strains might provide useful information about the circulation and a source of epidemiologically significant clones of *V. cholerae* on a global scale. In our study, we present the initial steps performed to obtain and process the whole-genome sequencing data of five *V. cholerae* O1 El Tor clinical isolates that were collected in the Russian Federation from imported cholera cases.

The clinical isolates were identified as *V. cholerae* O1 El Tor on the basis of standard biochemical and serologic testing. All studied isolates, RND6878, RND19191, RND1889, RND19188, and RND19187, represent cases of cholera imported from India. Two of them, RND19188 and RND19187, were isolated from a woman and her 10-month-old daughter.

Genomic DNA was extracted using the standard phenol-chloroform extraction protocol. Libraries for paired-end sequencing were prepared from 1 to 5 µg of genomic DNA by nebulization. Two steps of size selection were applied to get the libraries, with an approximately 500- to 700-bp insert size. The libraries were sequenced on the MiSeq platform using reagent kit version 2 (Illumina; San Diego, CA, USA). Raw sequencing data were processed by CASAVA1.8 software (Illumina; San Diego, CA, USA). The resulting raw reads were submitted to the NCBI Short Read Archive. To exclude low-quality data and adapter sequences for further analysis, reads were filtered out by Trimmomatic (1). Reads containing at least one base with a quality score of <20 were removed. The average insert size distribution estimated by PICARD tools (http://picard.sourceforge.net) varied in a narrow range, with a median of 500 bp. *De novo* assembly was performed using default options with CLC Genomics Workbench version 5.1 (CLC bio; Aarhus, Denmark) and SPAdes 3.0.0 (2). As a result, 164 and 234 contigs were generated, with estimated sizes of the genomes ranging between 3,991,231 and 4,005,480 bp. BLASTn (3) searches against the nonredundant nucleotide database were performed, and taxonomy assignments were manually validated to exclude potential contaminating sequences from the resulting set of contigs. The average coverage for each resulting draft genome varied between 88× and 136×.

It was found that isolate RND19191 is closely related to a group of Haitian isolates on the basis of identified core single nucleotide polymorphisms (SNPs) using a read mapping approach (4) of globally representative *V. cholerae* O1 El Tor strains (4–8).

A detailed report of the travel histories from cholera case patients as well as the results of comparative and phylogenetic analyses of these genomes and other available *V. cholerae* genomes will be published elsewhere.

### Nucleotide sequence accession numbers.

This whole-genome shotgun project has been deposited at DDBJ/EMBL/GenBank under the accession no. AYNL00000000 (RND6878), JNGT00000000 (RND19191), JNGU00000000 (RND19188), AYNM00000000 (RND19187), and AYNN00000000 (RND18899).
